# Serum ferritin, an early marker of cardiovascular risk: a study in Chinese men of first-degree relatives with family history of type 2 diabetes

**DOI:** 10.1186/s12872-019-1068-5

**Published:** 2019-04-03

**Authors:** Jun-Ru Liu, Yang Liu, Fu-Zai Yin, Bo-Wei Liu

**Affiliations:** 1 Department of Endocrinology, Qinhuangdao First Hospital, No.258 Wenhua Road, Qinhuangdao, 066000 Hebei Province China; 2 Department of General Surgery, Qinhuangdao First Hospital, No.258 Wenhua Road, Qinhuangdao, 066000 Hebei Province China

**Keywords:** Type 2 diabetes, Serum ferritin, Cardiovascular risk factors, Insulin resistance, Metabolic syndrome

## Abstract

**Background:**

Ferritin is one of the key proteins that regulate iron homeostasis and is widely available clinical biomarker of iron status. This study aimed to discuss the influence of serum ferritin (SF) on cardiovascular risk factors in the first-degree relatives with family history of type 2 diabetes (FHD).

**Methods:**

This cross-sectional study included 232 men. Anthropometric measurements and blood samples were analyzed. The people were divided into four groups according to median SF (102.8 ng/ml) and people with or without FHD. Group A (FHD–and low SF), group B (FHD–and high SF), group C (FHD+ and low SF), and group D (FHD+ and high SF).

**Results:**

The subjects in different categories of SF concentrations showed significant differences in BMI (SF main effect: *P* = 0.010), WC (*P* = 0.030), SBP (*P* < 0.001), FPG (*P* < 0.001), PPG-2 h (*P* < 0.001), FINS (*P* < 0.001), and HOMA-IR (*P* = 0.015; all: 2-way ANOVA). There was a significant difference in SBP (FHD main effect: *P* = 0.003), DBP (*P* = 0.006), and FINS (*P* = 0.013, all: 2-way ANOVA) between the groups with or without FHD. The interaction term between SF and FHD was significant for SBP (*P* = 0.011), DBP (*P* = 0.012), and PPG-2 h (*P* = 0.022). Logistic analysis showed that accumulation of CVD risk factors, which were ≥ 2 items and ≥ 3 items in group D were 7.546 and 3.343 times higher compared with group A (*P* < 0.05).

**Conclusions:**

The increased SF levels increased the risk of cardiovascular risk factors and the occurrence of insulin resistance in first-degree relatives with FHD.

## Background

The impact of cardiovascular disease (CVD) and type 2 diabetes mellitus (T2DM) has been increasing over the last decade, and its incidence is estimated to double the present rate by 2025. A family history of type 2 diabetes (FHD) is considered to be a major risk factor for CVD. Genetic factors are important in determining the affected adults. Our research revealed that adolescents with a positive FHD presented signs of insulin resistance (IR) and endothelial dysfunction [1, 2].

Metabolic syndrome (MetS) consists of a group of irregularly aggregated metabolic components with clinical characteristics of obesity, abnormally regulated glucose, metabolic disturbance in blood lipids and hypertension. IR is a common pathological and physiological feature of MetS [3]. The components of MetS, which are also considered as cardiovascular risk factors, directly promote the occurrence of atherosclerosis. MetS is associated with the cause of CVD that is independent of IR. Also, the combined effect of MetS and IR contributed to the risk of CVD [4]. The prevalence of CVD increases significantly with increased or aggravated abnormal metabolic components [5].

Ferritin is one of the key proteins that regulate iron homeostasis and is a widely available clinical biomarker of iron status. Some studies suggested that the prevalence of atherosclerosis and IR is significantly increased with increasing serum ferritin (SF) levels. Measurement of body iron store involves the use of many variables, wherein SF has been found to be a reliable tool by excluding the confounding effects of inflammatory, hepatic, or neoplastic diseases [6]. SF levels are independent predictors of IR in the evaluated male subjects [7]. The association of SF with CVD is still controversial. Previous studies have reported that increased SF in adults is closely associated with MetS and risk factors of CVD [8]. Recent meta-analysis showed that increased ferritin levels were independently and positively associated with the presence of MetS, with an odds ratio of higher than 1.73 [9]. However, a recent large sample study from UK shown that low iron status was associated with CVD risk in T2DM, suggesting that low iron status seems to be harmful for cardiovascular health in T2DM [10].

Non-traditional serum biomarkers of cardiovascular risk factors would contribute to explain this increased morbidity. However, the relationship of body iron store and individuals with cardiovascular risk and FHD remains to be investigated. Therefore, this study aimed to explore the relationship between SF and cardiovascular risk factors in the first-degree relatives with FHD.

## Methods

### Subjects

Healthy men (aged 50.6 ± 10.3 years) in Qinhuangdao, Hebei province during 2011 were enrolled. The study subjects were evaluated using a self-administering questionnaire to determine FHD. The standard for first-degree relatives of patients with type 2 diabetes included their parents, siblings or children. The inclusion criteria were as follows: 1) subjects who were clinically stable with no previous medical history of diabetes, hypertension, dyslipidemia, coronary artery diseases, or cerebral stroke; 2) subjects without clinical evidence of endocrinopathy; 3) subjects who were not taking medications that affect glucose and lipid metabolism, such as statins, glucocorticoids, thyroid hormones, and thiazide diuretics; 4) subjects were included if there was no signs of renal dysfunction, defined as serum creatinine > 115umol/l; 5) subjects were included if there was no signs of hepatic dysfunction, defined as > 1.5-fold elevation of alanine aminotransferase, aspartate aminotransferase. Subjects with anemia, who underwent blood transfusion recently, recent use of iron, smokers, alcohol, and with acute and chronic inflammation were excluded. This study was approved by the ethics committee of Qinhuangdao First Hospital. All subjects provided written informed consent before study initiation.

### Anthropometric measurements

Anthropometric measurements, including height, weight, waist circumference (WC), and blood pressure were obtained when the subjects were in light clothing and no shoes. Body mass index (BMI) was calculated by dividing weight (kg) by height squared (m^2^). Blood pressure was measured twice by using a mercury sphygmomanometer after 10 min of rest, while the subjects were seated, and the average of the two measurements was used for analysis.

### Laboratory examinations

All subjects underwent oral glucose tolerance test (OGTT) with 75 g of oral anhydrous glucose at 8:00 AM after 8 h of fasting. This was followed by dissolving 75 g of anhydrous glucose in 250 ml water. Peripheral venous blood samples were taken at 0 and 120 min after glucose loading. Plasma glucose concentration was measured using the glucose oxidase method, and serum lipid levels were measured using an enzymatic assay with an autoanalyzer (Hitachi, Tokyo, Japan). The plasma concentrations of fasting insulin (FINS) were measured by enzyme linked immunosorbent assay (ELISA) by using a model 680 microplate reader (BIO-RAD). ELISA kits were purchased from USCNLIFE company (intra-assay < 3% and inter-assay < 4%). The plasma concentrations of SF were measured by electrochemical luminescence with model Elecsys 2010 immunity analyzer (Roche, Germany). The kits were purchased from Roche company, Germany (intra-assay < 3% and inter-assay < 5%). The following equation was used to calculate the homeostasis model assessment (HOMA)-IR index: (fasting insulin level x fasting glucose level) / 22.5 and (HOMA)-βindex: 20 x fasting insulin level / (fasting glucose level - 3.5).

### Definition of groups

Cardiovascular risk factors were as follows: 1) abdominal obesity, which is defined as the presence of WC ≥ 90 cm in men; 2) impaired glucose homeostasis, which is defined as the fasting plasma glucose (FPG) ≥ 5.6 mmol/L and/or 2 h plasma glucose (PPG-2 h) ≥ 7.8 mmol/L; 3) Dyslipidemia, serum triglycerides (TG) ≥ 1.7 mmol/L, and/or serum high-density lipoprotein cholesterol (HDL-C) < 0.9 mmol/L in men; and 4) blood pressure ≥ 130/85 mmHg. Subjects were considered to have clustering of cardiovascular high-risk factors when they had at least two of the above-mentioned four traits.

The median (IQR) of SF was 102.8 (76.80, 162.04) ng/ml. The sample was divided into four groups according to the median of SF (102.8 ng/ml) and people with or without FHD. It was defined as low SF when SF was< 102.8 ng/ml, and high SF when SF was ≥102.8 ng/ml. The group A (FHD^−^and low SF) consisted of 59 subjects (age 49.6 ± 11.2 years), group B (FHD^−^ and high SF) consisted of 57 subjects (age 51.2 ± 11.7 years) without FHD and high SF, group C (FHD^+^ and low SF) consisted of 60 subjects (age 50.4 ± 11.3 years) with FHD and low SF, and group D (FHD^+^ and high SF) consisted of 56 subjects (age 50.0 ± 10.8 years) with FHD and high SF.

### Statistical analyses

All analyses were performed using SPSS 14.0 statistical software (SPSS 14.0 for Windows; SPSS, Inc., Chicago, IL). Values were expressed as mean with standard deviation. The count data was expressed as percentages. When not normally distributed, the data were presented as natural logs (ln)-transformed for analysis and are expressed as medians with interquartile ranges. Two-way analysis of variance (ANOVA) was performed to assess the main effect of SF and FH and the interaction. Pearson chi-square test was performed to compare the accumulation of CVD risk factors per group. Classification of risk analysis was based on multiple logistic regression analysis. *P* < 0.05 was considered to be statistically significant.

## Results

Age, anthropometric and biochemical data, are presented in Table 1. The age was similar in the four groups, with no significant difference (*P* > 0.05). Significant differences in general characteristics across different phenotypes of SF concentrations and FHD were searched by using two-way ANOVA (low SF versus high SF and adults with a family history of T2DM versus adults without family history of T2DM), and the main effects of SF and FHD and SF × FHD interaction were tested. Subjects characteristics with or without FHD and by different categories of SF concentration are shown in Table 1. The subjects in different categories of SF concentrations showed significantly different BMI (SF main effect: *P* = 0.010), WC (*P* = 0.030), SBP (*P* < 0.001), FPG (*P* < 0.001), PPG-2 h (*P* < 0.001), FINS (*P* < 0.001), and HOMA-IR (*P* = 0.015; all: 2-way ANOVA), whereas no significant difference was observed for age (*P* = 0.718), DBP (*P* = 0.725), TG (*P* = 0.975), HDL-C (*P* = 0.084), and HOMA-β (*P* = 0.891). A significant difference in SBP (FHD main effect: *P* = 0.003), DBP (*P* = 0.006), and FINS (*P* = 0.013, all: 2-way ANOVA) between the groups with and without FHD was observed, whereas no significant difference was found for age (*P* = 0.917), BMI (*P* = 0.165), WC (*P* = 0.213), FPG (*P* = 0.348), PPG-2 h (*P* = 0.168), TG (*P* = 0.720), HDL-C (*P* = 0.359), HOMA-IR (*P* = 0.391), and HOMA-β (*P* = 0.494). The interaction term between SF and FHD showed significant differences for SBP (*P* = 0.011), DBP (*P* = 0.012), and PPG-2 h (*P* = 0.022).

**Table 1:** Clinical and laboratory characteristics of the subjects in different groups

Variable						*P*	
	Group A *n* = 59	Group B *n* = 57	Group C *n* = 60	Group D *n* = 56	SF	FH	SF*FH
age (y)	49.61 ± 11.16	51.21 ± 11.71	50.44 ± 11.28	50.03 ± 10.81	0.718	0.917	0.541
BMI (kg/m^2^)	23.73 ± 2.64	25.34 ± 2.78	24.71 ± 4.21	25.83 ± 3.39	0.010	0.165	0.643
WC (cm)	86.50 ± 9.76	91.16 ± 10.20	89.48 ± 12.30	92.67 ± 11.13	0.030	0.213	0.680
SBP (mmHg)	113.0 ± 16.68	132.16 ± 22.73	129.67 ± 17.43	133.43 ± 21.27	< 0.001	0.003	0.011
DBP (mmHg)	82.19 ± 9.32	78.63 ± 9.17	82.59 ± 9.49	87.29 ± 11.81	0.725	0.006	0.012
FPG (mmol/L)	5.86 ± 0.63	6.51 ± 0.87	5.83 ± 0.81	6.77 ± 0.75	< 0.001	0.348	0.239
PPG-2 h (mmol/L)	7.92 ± 2.38	8.81 ± 2.61	7.47 ± 2.61	10.49 ± 3.61	< 0.001	0.186	0.022
FINS (uIU/ml)	9.22 ± 3.42	13.10 ± 5.67	10.09 ± 2.83	15.19 ± 7.85	< 0.001	0.013	0.498
TG (mmol/L)(IQR)	1.53(0.51,6.63)	1.61(0.67,6.63)	1.20(0.51,11.89)	1.79(0.50,10.52)	0.975	0.720	0.939
HDL-C (mmol/L)	1.36 ± 0.32	1.27 ± 0.37	1.31 ± 0.33	1.23 ± 0.67	0.084	0.359	0.919
HOMA-IR	0.35 ± 0.19	0.52 ± 0.24	0.40 ± 0.16	0.60 ± 0.23	0.015	0.391	0.843
HOMA-β	1.87 ± 0.19	1.90 ± 0.30	1.94 ± 0.16	1.93 ± 0.22	0.891	0.494	0.785
SF (ng/ml) (IQR)	72.90 (49.95,93.75)	151.95 (116.60,219.88)	79.92 (60.90,91.57)	163.90 (129.98,224.50)			

Values are expressed as means ± SD, TG and SF were non-normally distributed, they were ln-transformed for analysis and are expressed as medians (IQR). FINS, HOMA- β and HOMA-IR were non-normally distributed, they were ln transformed for analysis. Two-way ANOVA was performed to assess the main effect of SF and FH and the interaction between them. Group A consisted of 59 low SF adults without family history; group B consisted of 57 high SF adults without family history; group C consisted of 60 low SF adults with family history; group D consisted of 56 high SF adults with family history. BMI: body mass index; WC: waist circumference; SBP: systolic blood pressure; DBP: diastolic blood pressure; FPG: fasting plasma glucose; PPG-2 h: postprandial plasma glucose 2 h; FINS: fasting insulin; TG: triglycerides; HDL-C: high density lipoprotein cholesterol; HOMA: homeostasis model assessment; SF: serum ferritin; FH: family history

Pearson chi-square test was done to compare the accumulation of CVD risk factors per group in Table 2. There was significant difference in the detection rate of pathoglycemia and dyslipidemia among four groups (*P* < 0.05). There was no significant difference in the detection rate of abdominal obesity and hypertension among the four groups (*P* > 0.05). There was significant difference in the detection rate of cardiovascular risk factors components, which are ≥2 items and ≥ 3 items among four groups (*P* < 0.05). Logistic analysis showed that after adjusting of age, accumulation of CVD risk factors that are ≥2 items and ≥ 3 items in group D were 7.546 (95% CI: 2.166–26.291, *P* = 0.002) and 3.343 (95% CI: 1.407–7.944, *P* = 0.006) times higher compared with group A. (Table 3).

**Table 2:** Pearson chi-square test for comparison of accumulation of CVD risk factors per group

Variable	Abdominal obesity n(%)	High blood pressure n(%)	High blood glucose n(%)	Dyslipidemia n(%)	Cardiovascular risk factors for accumulation n(%)	
					≥2 terms	≥3 terms
Group A *n* = 59	25(42.4%)	12(20.3%)	22(37.3%)	4(6.8%)	26(44.1%)	13(22.0%)
Group B *n* = 57	33(57.9%)	12(21.1%)	32(56.1%)	15(26.3%)	35(61.4%)	19(33.3%)
Group C *n* = 60	25(41.7%)	12(20.0%)	19(31.7%)	9(15.0%)	29(48.3%)	11(18.3%)
Group D *n* = 56	32(57.1%)	14(25.0%)	37(66.1%)	15(26.8%)	41(73.2%)	26(46.4%)
*X* ^*2*^	6.156	0.698	26.904	10.499	17.244	14.495
*P*	0.104	0.874	< 0.001	0.017	0.001	0.002

**Table 3:** Logistic regression analysis for cardiovascular risk factors

Model	Single factor					Multiple			
		Wald	*P*	OR	95%CI	Wald	*P*	OR	95%CI
Cardiovascular risk factors for gathering≥2 terms	Group A			1.000				1.000	
	Group B	3.955	0.046	3.500	1.025–11.956	2.699	0.100	2.914	0.813–10.438
	Group C	0.021	0.886	0.938	0.388–2.267	0.198	0.657	0.807	0.314–2.076
	GroupD	9.706	0.002	6.750	2.030–22.440	10.072	0.002	7.546	2.166–26.291
	Age					7.416	0.006	1.053	1.015–1.094
Cardiovascular risk factors for gathering≥3 terms	Group A			1.000				1.000	
	Group B	1.043	0.307	1.625	0.640–4.125	0.262	0.609	1.292	0.485–3.438
	Group C	0.572	0.450	0.711	0.294–1.722	1.083	0.298	0.610	0.241–1.546
	GroupD	7.169	0.007	3.087	1.353–7.047	7.468	0.006	3.343	1.407–7.944
	Age					11.382	0.001	1.056	1.023–1.090

Group A: low SF adults without family history; group B: high SF adults without family history; group C:low SF adults with family history; group D:high SF adults with family history. Multiple logistic regression analysis:adjusted for age

## Discussion

Healthy offsprings of T2DM patients were often considered as models for studying early metabolic aberrations in the development of Type 2 diabetes. Genetic predisposition of type 2 diabetes also accelerates the development of atherosclerosis and potentially increases the risk of coronary heart disease. Previous studies have reported that healthy adults with FHD exhibited a significant damage in beta cell function [11]. Our results showed that the subjects in different categories of SF concentrations (SF main effect) had significantly different BMI, WC, SBP, FPG, PPG-2 h, FINS, and HOMA-IR. This suggested that the increased SF levels are associated with abdominal obesity, abnormal glucose metabolism, IR and elevated SBP. There was a significant difference in SBP, DBP, and FINS between the groups with FHD or without FHD (FHD main effect). This suggested that increased blood pressure and FINS are associated with FHD. Meanwhile, we speculated that the positive FHD increased the damage of islet function, leading to further increase of FINS and abnormal blood pressure. The interaction term between SF and FHD was significant for SBP, DBP, and PPG-2 h. Abnormal blood pressure and increased postprandial blood glucose are more obvious when there is a positive family history of diabetes and high SF levels at the same time. In our study, there were significant differences in the detection rate of cardiovascular risk factors components with ≥2 items and ≥ 3 items among the four groups. Logistic analysis after adjustment for age, showed that accumulation of CVD risk factors with ≥2 and ≥ 3 items in group D was 7.546 and 3.343 times higher compared with that in group A. The results showed that elevation of SF levels significantly increased the risk of cardiovascular risk factors together with a family history of diabetes.

Iron is an essential mineral in normal physiological processes, and ferritin is a specialized iron storage protein that reflects iron stores in the body [12]. A cross-sectional study conducted among 123 men showed that the SF levels are independent predictors for IR, omitting the effect of BMI and WC [7]. As known to all, IR is an important pathological basis for MetS and is associated with CVD. Excess body iron can impose oxidative injury that is associated with several cardiovascular risk factors including dyslipidemia, IR, and inflammation [13]. SF is considered as a reliable tool, providing that the confounding effects by inflammatory, hepatic, or neoplastic diseases are excluded [6]. It has been used as a surrogate variable to reflect the body iron stores in healthy individuals. Some recent researches from Asia shown that elevated SF levels may be a risk factor for T2DM [14], IR, MetS [15], and CVD [16]. It’s also been observed in our study that increased SF levels are associated with abdominal obesity, abnormal glucose metabolism, IR and elevated SBP. In contrast, a recent large sample study from UK shown that low iron status was associated with CVD risk in T2DM, suggesting that low iron status seems to be harmful for cardiovascular health in T2DM. However, the underlying mechanisms are still unclear [10]. The European Prospective Investigation into Cancer and Nutrition-Potsdam study concluded that high SF levels are associated with high risk of T2DM that is independent of established diabetes risk factors [17]. SF levels are often elevated in MetS or associated with a true hepatic iron overload. Elevated iron stores are associated with increased risk of T2DM in a Mediterranean population who are at high risk of CVD, after adjusting for fasting glucose and other components of the MetS. Similar association was not evident with soluble transferrin receptor (sTfR) [18]. sTfR levels could be spuriously elevated in subjects with IR and showed no association with MetS or its components [19]. A recent meta-analysis study conducted to evaluated the associations between ferritin levels, MetS and its individual components, and the potential role of confounding, showed that high triglycerides and glucose are the components that are more strongly associated with ferritin. Hepatic injury and BMI influenced the association of ferritin-MetS, and a threshold effect of high ferritin concentration on ferritin-high triglycerides association was observed [20]. The correlation between high triglycerides and ferritin is stronger, which was not observed in our study. This might be due to small sample size in our study. The difference in the detection rates of abdominal obesity and hypertension between the four groups showed no statistically significance, which was also related to the small sample size, and needs further confirmation by larger sample size study.

A recent study revealed that inflammatory cytokines may interact with MetS, obesity, IR, and T2DM [21]. Elevated SF levels may comprise an inflammatory state like that of MetS. There are plenty evidences regarding a relevant relationship between SF levels and inflammation, and SF is considered as an acute phase reactant and increased during inflammation [22]. And chronic inflammatory reaction may be considered to play an important role in the development and progression of IR. More studies are warranted to confirm this mechanism as it is becoming increasingly evident that excess iron is related to the incidence of MetS [23].

Our study was a cross-sectional study. The limitations should be considered when interpreting our results. We cannot determine the causal relationship between SF and cardiovascular risk factors. Experimental and prospective studies are warranted to elucidate the role of SF in the risk factors associated with CVD in the first-degree relatives with FHD. Although several reports were put forwarded regarding the relationship between SF levels and MetS [15, 16, 23], there has been no direct evidence whether SF is a predictor of accumulation of cardiovascular risk factors or not. In addition, small sample size and lack of data on women are also considered as limitations of this study. Therefore, a longitudinal relationship study between SF levels and accumulation of cardiovascular risk factors in larger samples size is warranted to confirm these results. Further studies are needed to confirm whether the correlation between SF and accumulation of cardiovascular risk factors also exists in women.

## Conclusion

In conclusion, elevated SF level increased the risk of cardiovascular risk factors in first-degree relatives with family history of diabetes in Chinese men. The increased SF elevated the occurrence of IR and MetS in first degree relatives with family history of diabetes and should be further investigated in future studies.

## Abbreviations

ANOVA: Analysis of variance
BMI: Body mass index
CVD: Cardiovascular disease
DBP: Diastolic blood pressure
ELISA: Enzyme linked immunosorbent assay
FHD: Family histoy of type 2 diabetes
FINS: Fasting insulin
FPG: Fasting plasma glucose
HDL-C: High-density lipoprotein cholesterol
HOMA: Homeostasis model assessment
IR: Insulin resistance
LDL-C: Low-density lipoprotein cholesterol
LSD: Least significant difference
MetS: Metabolic syndrome
OGTT: Oral glucose tolerance test
PPG-2 h: 2 h plasma glucose
SBP: Systolic blood pressure
SF: Serum ferritin
sTfR: Soluble transferrin receptor
T2DM: Type 2 diabetes mellitus
TG: Triglycerides
WC: Waist circumference
